# Thermometers

**Published:** 1880-02

**Authors:** 


There should be a thermometer in every chamber in the house, one in each hall or passage, and a large one at some easily accessible northern exposure out of doors, with a red column, and which could be seen without opening a door or window; they should be hung about five feet from the floor, not only for the purpose of enabling the children to see the index, but as indicating the temperature of the air which is breathed, as that at the floor is coldest, while that at the ceiling is the most heated as well as the most impure. With these facilities we can tell accurately whether our apartments are of a proper temperature; and also whether to put on more and heavier or lighter garments in the morning. By attention to these things we will save ourselves time and suffering, and many a doctor's bill, one of which would supply every room in the house with these useful articles, which, when once purchased, last for life if taken care of.

Speaking of changing the clothing, we consider it hazardous to lessen its amount after dressing in the morning, unless active exercise is taken immediately. No under-garment should be changed for lighter ones during the day ordinarily. The best safest and most convenient time for lessening the clothing, is in the morning when we first dress for the day. Hence, the first thing after rising should be to notice the thermometer. If you have but one, place it outside before getting into bed. Not less than twenty degrees from the temperature of the preceding morning should justify any special change in the clothing, unless persons are very sensitive.

There is a moral advantage in thermometers which merits the attention of every parent. All children love novelty, which is nothing less than knowledge to them, and they will take as much interest in what is usefully true, as in what is viciously so. You have only to turn their attention in a kindly encouraging and judicious way to the rise and fall of the mercury, and keeping a memorandum of it, in order to insure to them agreeable employment for many an hour in the year, and to consequent reflection, which we all know is the first step towards manliness and distinction. Make a child reflective, and he is safe for life. Get your children interested in observing natural truths, and you will have but little trouble in keeping them out of street associations, so that the purchase and proper use of a fifty cent thermometer may be to any child the difference between a life of disease and viciousness, or one of health and virtue, the difference between a life lost and a man saved to his country and his race.

